# Transforming a General Hospital to an Infectious Disease Hospital for COVID-19 Over 2 Weeks

**DOI:** 10.3389/fpubh.2020.00382

**Published:** 2020-07-28

**Authors:** Navin Pandey, Vipin Kaushal, Goverdhan Dutt Puri, Sunil Taneja, Manisha Biswal, Pranay Mahajan, Rashmi Ranjan Guru, Pankaj Malhotra, Inderpaul Singh Sehgal, Sahajal Dhooria, Valliappan Muthu, Ritesh Agarwal

**Affiliations:** ^1^Department of Hospital Administration, Postgraduate Institute of Medical Education and Research, Chandigarh, India; ^2^Department of Anesthesia and Critical Care, Postgraduate Institute of Medical Education and Research, Chandigarh, India; ^3^Department of Hepatology, Postgraduate Institute of Medical Education and Research, Chandigarh, India; ^4^Department of Medical Microbiology, Postgraduate Institute of Medical Education and Research, Chandigarh, India; ^5^Department of Internal Medicine, Postgraduate Institute of Medical Education and Research, Chandigarh, India; ^6^Department of Pulmonary Medicine, Postgraduate Institute of Medical Education and Research, Chandigarh, India

**Keywords:** hospital management, infection control, isolation unit, infectious disease hospital, COVID-19

## Abstract

Pandemics like the coronavirus disease (COVID)-19 can cause a significant strain on the healthcare system. Healthcare organizations must be ready with their contingency plans for managing many patients with contagious infectious disease. Ideally, every large hospital should have a facility that can function as a high-level isolation unit. An isolation unit ensures that the healthcare staff and the hospital are equipped to deal with infectious disease outbreaks. Unfortunately, such facilities do not exist in several hospitals, especially in resource-limited settings. In such a scenario, healthcare setups need to convert their existing general structure into an infectious disease facility. Herein, we describe our experience in transforming a general hospital into a functional infectious disease isolation unit.

## Introduction

An outbreak of pneumonia of unknown origin was reported from the Wuhan province of China in December 2019. Subsequently, the pneumonic illness was found to be caused by a novel coronavirus (CoV) resembling the severe acute respiratory syndrome (SARS) virus (1). The World Health Organization (WHO) named the virus as SARS-CoV-2 and the disease as Coronavirus Disease 19 (COVID-19) (2). Further, the disease was declared as a pandemic by the WHO on 11 March 2020 (3). Worldwide experience shows that the interventions of “social distancing,” extensive testing, and isolation of patients has helped in flattening the epidemiological curve (4). Simultaneously, the healthcare facilities (including critical care units) have to be up-scaled to handle the severely ill COVID-19 patients.

The first case of COVID-19 in India was reported in January 2020 (5). The Government of India declared a nationwide closure on 24 March 2020 as a measure to contain the spread of this pandemic (6). Meanwhile, identifying various healthcare centers in different parts of the country as isolation and treatment centers for COVID-19 became a key component to address this challenge. Our center is a tertiary-care, teaching, and referral hospital in north India. We were designated as a dedicated COVID-19 hospital for our area.

While the ideal design and facilities for a high-level isolation unit have been clearly defined (7), the problem arises when such a facility is not available. In the wake of the COVID-19 crisis, we must improvise to create the best possible facility using available resources. There is guidance available for establishing an infectious disease unit (8); however, implementation is a daunting task and requires pragmatic solutions. In general, setting up a new facility involves four main components: space, stuff (supplies), staff, and maintaining standards (9). Herein, we describe our experience in transforming a general healthcare facility to a dedicated infectious disease setup in the wake of the COVID-19 pandemic. A brief overview of the health care infrastructure in India and response to the current pandemic is outlined in Table 1 (10–13).

**Table 1:** Brief overview of health care infrastructure in India and response to the COVID-19 pandemic.

**Health care infrastructure in India**
• Health is a state issue in India, and there are three tiers of public health system.
• Hospital services are provided by several public sector hospitals (sub-district, district, medical college hospitals, and institutes of national importance). However, private (corporate) sector clinics, nursing homes, and hospitals serve most of the population, particularly in the urban areas.
• India is committed to Universal Health Coverage scheme, and to date, 0.5 billion (one-third of the population) have benefited from this scheme. Apart from the national insurance scheme, several programs run by State governments and government organizations also strive toward the common goal of achieving equitable access to health care.
• India has a population of about 1.34 billion, and the densely populated cities and towns are at risk for infectious disease outbreaks due to resource constraints. Nearly 60% of the Indian population reside in rural areas; however, healthcare services are mainly concentrated in urban areas.
• Alternative medical systems practiced in India include Ayurveda, Yoga and Naturopathy, Unani, Siddha, and Homeopathy (AYUSH). The Department of AYUSH under the Central Ministry of Health and Family Welfare also promotes health and addresses the unmet health care needs.
• As per the National Sample Survey (NSS), there are an estimated 3.8 million healthcare professionals (including AYUSH professionals) as of January 2016. The density of doctors and nurses and midwives per 10,000 population is about 21. More than 80% of doctors and 70% of nurses and midwives are employed in the private sector.
**COVID-19 pandemic in India and the response from the health care sector**
• India is experiencing an increasing number of COVID-19 cases. Currently (17th June, 2020), there are 350,000 cases with about 11,900 deaths. The densely populated cities account for a large proportion of cases.
• The government has gone to great lengths to deal with the pandemic. The nation was in a state of “lockdown” for almost 2 1/2 months. During the lockdown, the health infrastructure was significantly up-scaled. For instance, the national government has ordered 50,000 ventilators.
• Both public and private sector hospitals are involved in COVID care. The COVID care in public sector hospitals is free of cost. The government has also rationalized the cost of COVID care in the corporate sector in several parts of the country.
• The Government of India has already established guidelines for the purpose of upscaling healthcare. An advisory has been issued to manage COVID-19 cases based on clinical severity. Mild or asymptomatic cases would be managed at home where there is social support. In those lacking social support, large-scale COVID care centers have been assigned for these purposes, including AYUSH hospitals. Moderately severe cases (pneumonia with no hypoxemia) will be managed at a dedicated COVID health center, and the severe cases would be admitted to COVID hospitals.
• For managing hospitalized COVID-19 patients, the departments have been categorized into category A (core departments like anesthesia, pulmonary and critical care, internal medicine, emergency medicine), B (clinical specialties already running an ICU such as cardiology, gastroenterology, Neurosurgery, Cardiac surgery, and others), C (clinical specialties not running ICU like endocrinology, rheumatology, orthopedics, obstetrics, and others), D (other specialties with limited or no responsibility for critical care like dermatology, ophthalmology, community medicine, and others), E (medically trained MBBS resident from pre- and para-clinical departments), and F (interns). Doctors from category A and B departments will handle critically ill patients, while non-critically ill patients will be handled by category C and D department with a team leader from category A or B. In exigent situations, nursing professionals, the third- and fourth-year nursing students would also have to be involved in healthcare delivery.
**Implications for future: lessons from the pandemic**
• Medical and nursing curricula should incorporate a section on disaster preparedness and management as a part of their training. The importance of dealing with epidemics and pandemics, especially of respiratory origin, should also be imparted in medical training.
• Hospital management training should include disaster management particularly up-scaling and diverting existing services and resources in the time of disaster.
• Infectious disease units with facilities for isolating contagious patients should be developed in tertiary hospitals across the country. Such units ensure that a dedicated unit with necessary facilities and, most importantly, trained workforce is available, which can be expanded as and when required.

### Administrative Setup of Our Center

Our Institute is a public sector tertiary care research hospital directly governed by the Ministry of Health and Family Welfare (India). The Director is the CEO equivalent of the Institute followed by the Dean. In the current pandemic, the Director constituted several committees, and the one for the COVID hospital was chaired by the Dean (GDP) with several members who took various decisions. The members were from the hospital administration (RRG, PM, NP, and VK), pulmonary medicine (VM, ISS, SD, and RA), infection control (MB), and internal medicine (ST and PM). The committee then brainstormed for 2 days and outlined the important initial tasks. The tasks were then completed in a time-bound fashion with the help of the hospital engineering, hospital procurement team, and countless other staff.

There were certain important policy decisions beyond this hospital. The decision for a separate COVID hospital was undertaken by the government of India such that the non-COVID essential patient care continues unimpeded. The non-essential services were, however, curtailed not only as an infection prevention measure but also to divert the resources toward effectively handling the pandemic. The essential services such as emergency, intensive care unit, bronchoscopy, and others were continued.

## Problem No. 1: Identification of a Dedicated Site For the Infectious Disease Hospital

**Figure 1:**
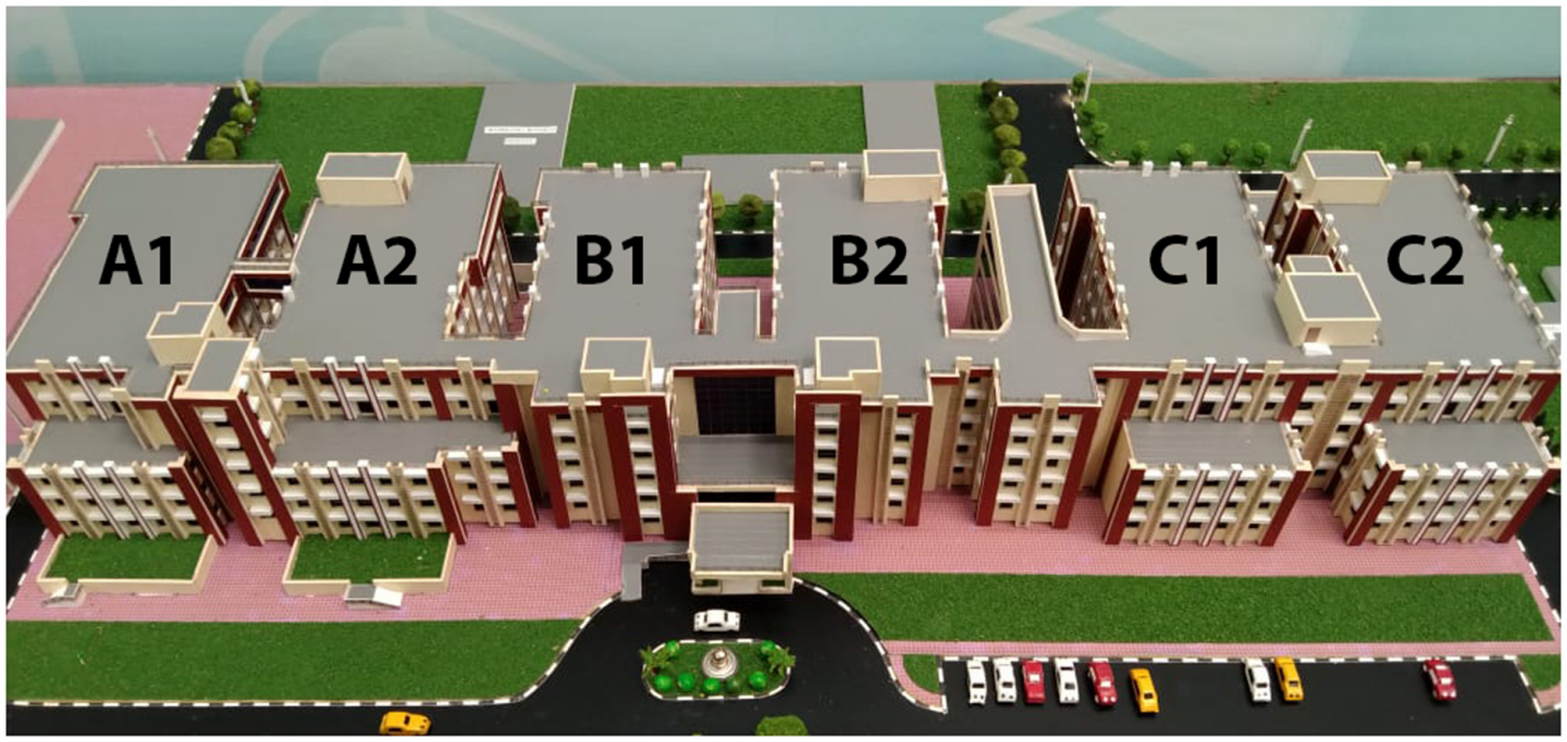
A model of the hospital transformed to function as COVID-19 facility. The design resembles a comb with the corridor representing the shaft and the blocks representing the comb's teeth. There are five levels and a basement.

The first challenge was identifying a dedicated site for managing COVID-19 patients. Ideally, a hospital block that can be separated from the main hospital should be identified (14). Else, an area that can be physically isolated from routine services is also acceptable (15). We were fortunate to have a ready-to-use 200-bed newly constructed hospital physically separate from the main hospital. Most situations will not have such good fortune; hence, reliance on temporary hospitals will be required (16–18). However, such temporary facilities can handle only mild to moderate cases and not those requiring intensive care. A few departments that had started functioning in the new building of our hospital were relocated to their original sites. The building is designed in the form of six rectangular blocks interconnected at one end through a common corridor (Figure 1). The design resembles a comb with the corridor representing the shaft and the blocks representing the comb's teeth. There are five levels and a basement. The intensive care units (ICUs), high dependency units (HDUs), and operating theaters (OTs) are housed at the fifth level (total capacity of 50 ICU/HDU beds). The fourth level consists of 50 independent rooms. The third and the second levels have general wards and a few independent rooms, with a total capacity of 100 patients. In total, the new hospital could cater to about 200 patients. The reception and administrative offices are located on the ground floor (Level 1). The basement has the facility for the central store (for equipment, drugs, linen, and others).

We decided that the fourth and fifth levels will be utilized first. Subsequently, with increasing patient numbers, we planned the utilization of the third and the second levels. The ICU and pre- and post-operative rooms (collectively labeled the HDU) on the fifth level were demarcated for critically ill patients, while single rooms on the fourth level were allocated for isolating relatively stable patients. A single OT on the fifth level was selected for performing any surgeries on COVID-19 patients.

### Practice Points

- Choose a separate building to function as an infectious disease facility, away from the non-COVID hospital. If such a facility is not available, identify an area that is physically disconnected from the general non-COVID area, with a separate entry and exit.

## Problem No. 2: Shifting of Equipment For Use in the COVID Hospital

The newly established hospital lacked facilities for managing critically ill patients, as the purchase of equipment was delayed owing to the lockdown. The departments of anesthesia and pulmonary medicine got together and made a checklist of requisites to run an ICU. We then used the various departments' existing inventory to stockpile the new ICU with the necessary supplies and medications. Subsequently, we quickly shifted the supplies from their respective areas for use in the COVID complex. These included ICU ventilators, defibrillators, monitors, crash carts, and other consumables. Further, we mobilized the portable electrocardiography and radiology machines, dialysis machines, and a point-of-care blood analyzer for use in the ICU. We implemented standard infection control practices, including the use of a separate stethoscope, thermometer, sphygmomanometer, and others in the patient care areas. One staff from each department was present throughout the initial week to mobilize the equipment, as required.

### Practice Points

- Create a checklist and mobilize the existing resources to initiate hospital services. A multidisciplinary team approach helps in the pooling of resources during a crisis.

## Problem No. 3: Streamlining the Movement of Patients and Healthcare Workers

**Figure 2:**
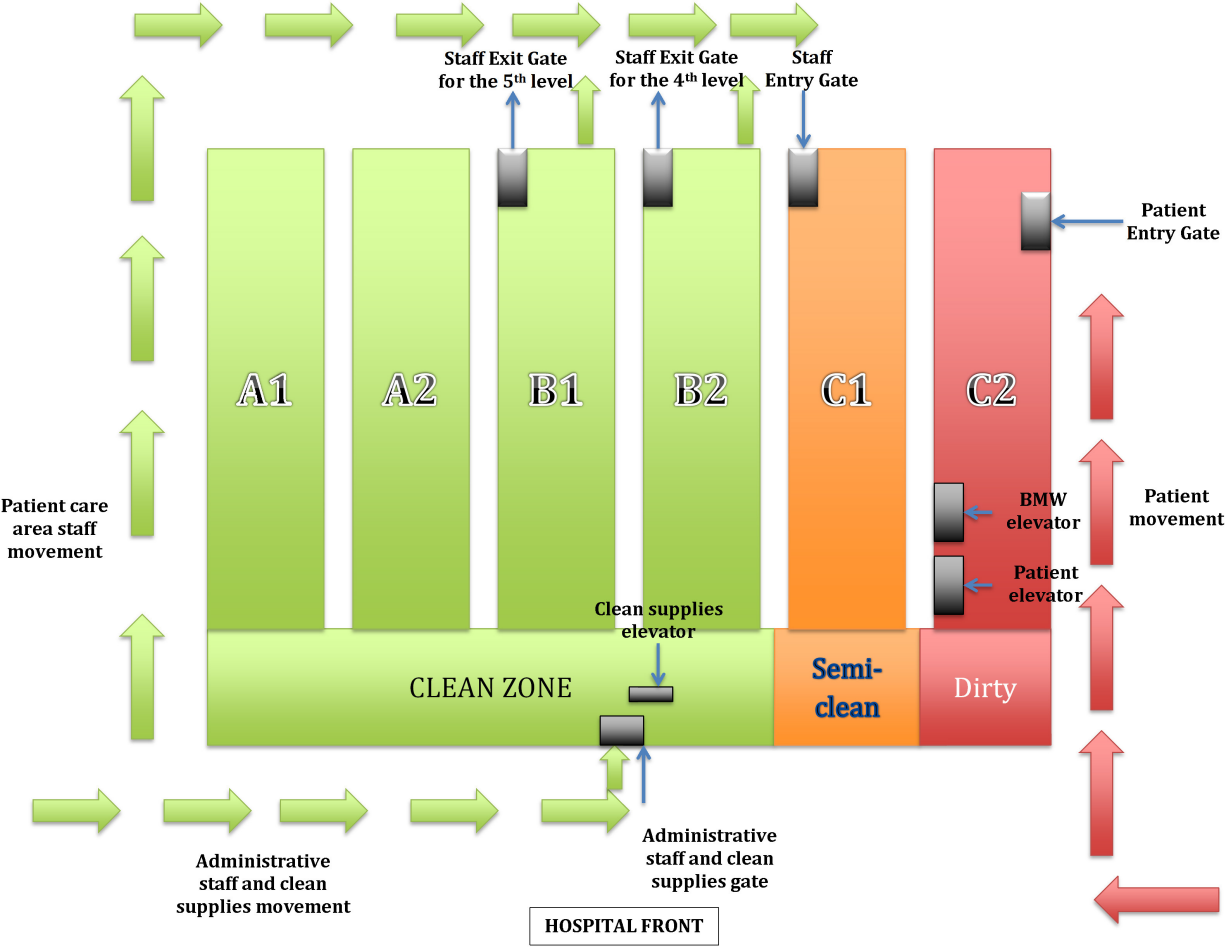
A schematic representation of the functioning of the transformed hospital. A clear demarcation between the clean and contaminated zone can be appreciated in the picture.

An important consideration was delineating the flow of patients and the hospital staff to ensure the demarcation of contaminated (dirty), semi-clean, and clean areas. We identified the hospital block at the farthest end of the hospital premises as the point of entry for patients (Figure 2, block C2). We identified a single elevator in that block for the movement of patients to different floors. A triage area was set up in this block near the entrance. The patient was transported from the triage area to the respective floors through a dedicated (“contaminated”) elevator (Figure 2). This entire zone was labeled a contaminated zone, and barriers were installed to guarantee no movement of staff or materials from this zone to the rest of the block on this level (Figure 2, block C2). We delineated a separate entry for the movement of the staff posted on different levels, thereby completely avoiding an intersection between the two paths. We dedicated a floor-to-floor inclined ramp for the transfer of patient samples in closed boxes; this area was designated as relatively clean. The staff movement zone was also considered a relatively clean zone. Finally, a clean zone was created on the ground floor (Level 1) with a single entry and exit through the main reception area, different from the two abovementioned zones. The clean area was meant for use by the staff not directly involved in patient care (administrative staff, material transfer, and others). An elevator identified in the clean area on level 1 was used for the transfer of clean materials to different levels without physically “getting down” on the respective floor.

### Practice Points

- Plan the infectious disease facility, taking care that the most infectious patients are kept away from the common entry points.
- We must ensure a clear boundary between contaminated, relatively clean, and clean zones.

## Problem No. 4: Identifying Areas For Donning and Doffing of Personal Protective Equipment (PPE)

We converted one office room into a space for storage, distribution, and audit of PPE. A store assistant was posted in this area to distribute PPE and assure rational utilization. We arranged a snack counter in this area for the staff who are to perform the ICU shifts, as the PPE had to be worn for the next 6 h. A common donning area consisting of individual rooms was created near the staff entrance area (Figure 2, block C1) by converting four office spaces into changing rooms for the male and the female staff. We installed full-length mirrors, placed chairs, and provided for hand hygiene in the donning rooms. We placed lockers in the area adjoining the donning room for the staff to secure their belongings, including personal mobile phones. We had a contingency plan wherein an entire corridor could be converted into a donning area once the number of patients increases, and more HCWs are required in the respective areas. An elevator (semi-clean) in this block was assigned for the staff to enter the fourth and fifth levels. The elevator was programmed to stop only on the fourth and fifth levels. The security personnel ensured a one-way movement of staff, i.e., from semi-clean to contaminated zone and not vice versa.

The doffing areas are the actual problematic zones, and meticulous planning is required while designing these areas. We identified two rooms, each on the fourth and the fifth levels, as doffing areas. These rooms were located near the fire exit staircases, farthest from the common corridor (other ends of each block), thereby ideally suited for doffing (Figure 2). The doffing rooms had two separate doors, one for entry and the other for exit. We partitioned the doffing area into clean and dirty zones, installed full-length mirrors, and placed hand sanitizers. We also installed exhaust fans to improve air circulation. Immediately after doffing, the staff had to exit through the fire exit staircases and make their way back through the same staff entry gate (Figure 2, block C1). We earmarked a few rooms for de-scrubbing on level 1, where the HCWs had to remove their scrubs and shower before changing into their usual clothes. The de-scrub rooms also had full-length mirrors, appropriate waste disposal bins, hand sanitizers, and masks. We also made provisions for light snacks at the exit point.

### Practice Points

- Identify and label all entry, exit, stairs, and escalators to prevent the intersection of clean and contaminated paths.
- Provide facilities for refreshment, shower, and lockers in the donning and de-scrub areas. This area should be separate from the contaminated zone.

## Problem No. 5: Air Conditioning and Air Distribution

The hospital was originally not meant for highly contagious infectious disease patients. The heating, ventilation, and air conditioning (HVAC) system of the building was designed for recirculation of air inside the premises. Moreover, the HVAC system had a capacity only for six air exchanges per hour and no provision for a negative pressure unit. We made the following modifications. We sealed the return ducts in the ICU and HDU areas to prevent the recirculation of air. Further, we installed exhaust fans in the ICU, HDU, and doffing areas to ensure at least 12 air exchanges per hour (19, 20). In places where the return ducts could not be sealed (like the corridors), the air-conditioning was switched off, and the smoke exhaust system was used every 2 h to push the air out and allow fresh air. Exhausts were present in the toilets of individual patient rooms. Additionally, the windows were kept open in patients' rooms and corridors to enhance natural ventilation.

### Practice Points

- Prevent recirculation of air and assure at least 12 exchanges per hour. Utilize low-cost systems such as simple exhaust fans for air exchanges.

## Problem No. 6: Lack of Trained Manpower to Work in an Infectious Disease Facility

We decided that the HCWs would work for 7 days. During the 7-day duty period, the staff was provided accommodation within the hospital campus. Quarantine facilities were created for these staff by vacating individual private rooms in a separate building of the hospital. The HCWs in this setup were expected to be in a PPE for the entire duration of their shift; hence, we restricted the duty to 6 h. However, these measures of limited duty periods and prolonged quarantine required many HCWs. Our Institute routinely caters to around 10,000 patients in the outpatient department every day. Given the ongoing nationwide lockdown, we curtailed the outpatient services, elective admissions, and surgeries as a measure to reduce overcrowding. The staff employed in all these services were allocated to the new facility. The new staff posed a challenge as the HCWs were inexperienced with the different protocols of a respiratory isolation unit. To overcome this problem, we trained the HCWs on an urgent basis. Several teams were created to impart training with the ICU protocols, infection control practices, including the donning and doffing of PPE, and the movement of staff, patients, and materials inside this new facility. The auxiliary staff delivering services like hospital engineers, security guards, dieticians, epidemiologists, and ambulance drivers were also trained in appropriate PPE and other relevant infection control protocols. We installed closed-circuit television (CCTV) cameras and public address systems in the doffing areas for real-time monitoring. A control room was activated to continuously monitor CCTV outputs of the clinical areas to identify any discrepancies and apprehend any breaches in the established protocols.

### Practice Points

- Curtail non-essential hospital services to reallocate the HCWs for dealing with the pandemic.
- Provide intensive training in the ICU and infectious disease practices for the newly recruited HCWs.
- Employ telemonitoring of the HCWs to ensure adherence to the laid down protocols.

## Problem No. 7: Arranging the Requisite Personal Protective Equipment

Since the onset of this pandemic, photographs, and videos of HCWs in hazmat suits had become commonplace. Most HCWs were convinced that this was mandatory for patient care. Initially, we did not have enough supplies to meet the anticipated demand for PPE. We decided on a PPE kit consisting of the following items namely, hospital scrubs, surgical cap, water-impervious surgical gown with a hood, an overall surgical gown (sterile gowns for the OTs, ICU, and HDU), protective goggles, N95 mask, visor, knee-length shoe cover, surgical mask, and two pairs of gloves. The procurement of PPE was a daunting task as the supply chain was interrupted owing to the nationwide lockdown. To maintain the supply chain, we decided to stockpile all the necessary PPE required for at least a month's duration. Fortunately, the textile companies responded to the government's plea and started the manufacture of various components of PPE. In fact, India is currently the second largest manufacturer of PPE worldwide and is considering export (21). We contacted local manufacturers and vendors. The samples from different vendors were evaluated by an expert committee comprising of a microbiologist, internist, pulmonologist, and hospital management. No single supplier could provide the products mentioned above of good quality in a single package. Hence, we purchased the individual components of the PPE from different manufacturers. A team of hospital staff then assembled the individual items in a single package. By the time we had 15 patients in this new setup, we were utilizing around 70 PPEs in a day consisting of four duty shifts.

### Practice Points

- In the absence of a supply of standard PPE, utilize local vendors to supply the individual items, which can then be assembled in a single package. However, one must ascertain that strict quality control measures are in place.
- Involve end-users in evaluating the appropriateness of PPE.

## Problem No. 8: Provision of Laboratory and Radiology Services

A portable machine for radiography and ultrasound machines were placed in the patient care areas. Technicians were trained in the donning and doffing process and were available on call. We mobilized a point-of-care arterial blood gas analyzer from another area for use in the ICU. We provided separate iceboxes in all clinical areas for the storage of virology swabs and other samples. The vacutainer tubes were stored in Ziplock bags in these icebox containers. We instructed all the staff in the clinical areas to have the icebox containers placed at the entrance of the inclined ramp identified for the transport of samples on the respective floors. A hospital attendant would reach the respective levels through the designated ramp (relatively clean zone). The attendant would then transfer the contents of the ward icebox to a clean icebox. The clean icebox would subsequently be transported to the sample collection area. The samples were transported to the respective laboratories by laboratory personnel at a fixed time every day. We used WhatsApp to implement a paperless request for investigations and receiving reports, to strengthen our infection control practice further.

### Practice Points

- It may not be possible to have the complete support of routine services like laboratory, radiology, and others within a new infectious disease setup. Therefore, develop simple protocols for handling the samples and conducting radiological investigations to minimize cross-contamination.

## Problem No. 9: Development of Standard Operating Procedures (SOPs)

Hospitals are typical matrix organizations with various departments and a blurred line of staff control (22). Hence, SOPs are integral for the functioning of any hospital (23). We developed SOPs for all the support services, as described below.

### Dietetics, Pharmacy, and Other Supplies

To minimize the exposure of hospital staff involved in the delivery of various materials, we undertook the following steps. A nursing officer in the clean zone was made responsible for coordinating and procuring all the materials required for patient care. We provided smart phones at the nursing stations of the patient care areas (contaminated) at each floor for easy communication with the coordinating nurse (clean) and other staff.

We used disposable cartons for the transfer of all materials to decrease the risk of infection. The boxes were transported to the respective floors through an elevator in the clean zone (Figure 2, level 1, block B2) by a hospital attendant. This elevator could only be operated from outside on the first level, thereby ensuring that no HCW from the contaminated zone could use the elevator to reach the ground floor (first level). The attendant would push the package outside the lift on the respective floor without stepping out. The cartons were then picked up by another hospital attendant on the floor and discarded after use from the patient care areas like other biomedical waste (BMW).

### Laundry

All linen from patient care areas were immersed in 1% sodium hypochlorite solution for a period of half an hour (24). The disinfected linen was then transported by a laundry attendant wearing PPE using an elevator adjoining the one used for patient movement in the contaminated area (the “contaminated elevator for BMW”).

### Biomedical Waste

We anticipated a substantial amount of BMW to be generated in every shift because of the extensive use of PPE by all staff involved in service delivery. We decided that all the generated waste would be collected in double bags and disinfected as per the current guidelines (25). Personnel donned in PPE from the BMW department would visit these areas in every shift with their transport trolley using the “contaminated elevator for BMW.”

### Discharge

The same route by which the patients enter was also used for discharge. COVID-19 had created a unique situation wherein, at times, no patient attendants are available at the time of discharge of patients. The social stigma associated with the disease compounded the problem further. Thus, we needed to drop these patients to their homes by ambulance.

### Death

After the demise of the patient, the body was wrapped in double body bags, and shifted to the mortuary by trained personnel donned in PPE. The local governing body undertook the responsibility of transportation from the hospital mortuary to the area dedicated to performing the last rites (26). The ambulance used to carry patients to the hospital or the dead bodies to mortuary was disinfected using 1% sodium hypochlorite between patients.

#### Practice Points

- Develop standard operating protocols for support staff to provide efficient and unhampered service.

## Problem No. 10: Psychological Support for the HCWs

We anticipated tremendous stress would occur on the HCWs involved in COVID-19 care for various reasons. These include the highly infectious nature of the illness, working in a new environment wearing the PPE, potential risk of infecting themselves and their loved ones, inability to cope with the uncertainties surrounding the pandemic, being distanced from their family during a week-long duty, and news reports of death amongst the HCWs. A dedicated team from the department of psychiatry provided psychosocial support to HCWs and their families on a round-the-clock basis. We established a 24-h helpline number to assist them in this task.

### Practice Points

- Tremendous stress is expected amongst the HCWs and their families during such pandemics. Extend psychosocial support that is readily available and accessible.

## Conclusions

Disasters, especially a pandemic like COVID-19, demand a sudden change in all pre-existing practices. The most crucial point that we have learned from this pandemic is the need for disaster preparedness. Every hospital needs to maintain a contingency of resources and have an active infection control and disease outbreak team in place. Before any hospital management, the challenge is to come up with solutions that are scientific, practical, and simple enough to be implemented and adopted by the multiple categories of staff in a hospital. We have provided a broad outline for transforming a general hospital into an infectious disease hospital. Importantly, the changes that we made to the hospital are temporary, ensuring that we can bounce back to normal quickly once the pandemic subsides. However, the lessons learned have long-lasting implications for policymakers.

## Data Availability Statement

The original contributions presented in the study are included in the article/supplementary material, further inquiries can be directed to the corresponding author/s.

## Author Contributions

NP, VK, GP, ST, MB, PMah, RG, PMal, IS, SD, VM, and RA drafted and revised the manuscript. All authors contributed to the article and approved the submitted version.

## Conflict of Interest

The authors declare that the research was conducted in the absence of any commercial or financial relationships that could be construed as a potential conflict of interest.
